# Genome size differentiates co-occurring populations of the planktonic diatom *Ditylum brightwellii *(Bacillariophyta)

**DOI:** 10.1186/1471-2148-10-1

**Published:** 2010-01-02

**Authors:** Julie A Koester, Jarred E Swalwell, Peter von Dassow, E Virginia Armbrust

**Affiliations:** 1 School of Oceanography, Box 357940, University of Washington, Seattle WA 98195-7940, USA; 2 CNRS, UMR7144, Evolution du Plancton et PaleoOceans, Station Biologique de Roscoff, BP 74, Roscoff 29682, France

## Abstract

**Background:**

Diatoms are one of the most species-rich groups of eukaryotic microbes known. Diatoms are also the only group of eukaryotic micro-algae with a diplontic life history, suggesting that the ancestral diatom switched to a life history dominated by a duplicated genome. A key mechanism of speciation among diatoms could be a propensity for additional stable genome duplications. Across eukaryotic taxa, genome size is directly correlated to cell size and inversely correlated to physiological rates. Differences in relative genome size, cell size, and acclimated growth rates were analyzed in isolates of the diatom *Ditylum brightwellii*. *Ditylum brightwellii *consists of two main populations with identical 18s rDNA sequences; one population is distributed globally at temperate latitudes and the second appears to be localized to the Pacific Northwest coast of the USA. These two populations co-occur within the Puget Sound estuary of WA, USA, although their peak abundances differ depending on local conditions.

**Results:**

All isolates from the more regionally-localized population (population 2) possessed 1.94 ± 0.74 times the amount of DNA, grew more slowly, and were generally larger than isolates from the more globally distributed population (population 1). The ITS1 sequences, cell sizes, and genome sizes of isolates from New Zealand were the same as population 1 isolates from Puget Sound, but their growth rates were within the range of the slower-growing population 2 isolates. Importantly, the observed genome size difference between isolates from the two populations was stable regardless of time in culture or the changes in cell size that accompany the diatom life history.

**Conclusions:**

The observed two-fold difference in genome size between the *D. brightwellii *populations suggests that whole genome duplication occurred within cells of population 1 ultimately giving rise to population 2 cells. The apparent regional localization of population 2 is consistent with a recent divergence between the populations, which are likely cryptic species. Genome size variation is known to occur in other diatom genera; we hypothesize that genome duplication may be an active and important mechanism of genetic and physiological diversification and speciation in diatoms.

## Background

Genotypic and physiological variation is frequently disguised by an apparent morphological constancy traditionally assumed to be stable enough for the assignment and identification of species. Cryptic species that display subtle variations in morphology associated with reproductive isolation have been described in all major phylogenetic lineages of eukaryotic marine phytoplankton [1-4], despite the fact that large population sizes and ocean mixing were expected to facilitate gene flow and homogenize species distinctions. Diatoms are the youngest [5] and the most species-rich group of phytoplankton [6,7]; they have risen quickly to become important contributors to oceanic ecosystems as primary producers and intermediates in the global biogeochemical cycles of carbon and silicon [8-10]. The mechanisms of speciation in diatoms remain under investigation.

Abrupt changes in an organism's genome size through polyploidy can lead to reproductive isolation and eventual speciation [11,12]. Diatoms are the only major group of eukaryotic phytoplankton with a diplontic life history, in which all vegetative cells are diploid and meiosis produces short-lived, haploid gametes, suggesting an ancestral selection for a life history dominated by a duplicated (diploid) genome. Polyploidization accounts for 2-4% of speciation events in flowering plants and up to 7% of speciation events in ferns [13]. In addition, stable polyploids were observed among laboratory populations of the diatom species *Thalassiosira weissflogii *(Grunow) Fryxell and Hasle [14]. Polyploidization may underlie the variation in chromosome number observed between and within diatom species [15-18].

A change in genome size precipitates a cascade of cellular responses leading to nearly universal relationships among genome size, cell size and metabolic rates [19,20]. In accord with other divergent taxa, genome size and cell size in phytoplankton are positively correlated [14,21-23]. Growth rates are inversely correlated with genome and cell sizes such that large-celled species with more DNA, including diatoms, grow more slowly than small-celled species with less DNA [24-26].

The relationship between cell size and genome size is of additional interest in diatoms. Asexual mitotic division produces two daughter cells, one of which is smaller than the mother cell due to the constraints of the rigid cell wall. Over time, the mean cell size of a clone decreases with each successive round of division, whereas the variance in size increases [27,28]. Large cell sizes are restored through sexual reproduction, or, less frequently, through asexual enlargement [29]. In a clonal lineage, the original sexual offspring can have 100-fold larger volumes than the smallest cells produced asexually. The smallest size that may be attained by a species is likely influenced by genome size, but the sizes of the largest cells are likely the result of genetic and environmental interactions during zygotic development.

High genetic divergence characterizes two co-occurring populations of the common coastal diatom *Ditylum brightwellii *(T. West) Grunow in van Heurk, which has a wide-spread coastal and estuarine distribution. In Puget Sound, WA, *D. brightwellii *is composed of two metapopulations that are defined by DNA sequence differences in the ribosomal internal transcribed spacers (ITS) [30,31]. Both metapopulations consist of two or more populations (defined by differences in microsatellite allele frequencies) that can co-occur in the water column [30-34]. For simplicity, the ITS-defined metapopulations will be referred to as populations throughout this study. Based on ITS sequencing of clones from the eastern and western margins of the Pacific and Atlantic Oceans including the Yellow Sea (Genbank: EU364892) and Gulf of Maine (pers. obs.), population 1 appears to have a circum-global distribution in temperate waters, while, to date, population 2 has been found only in Puget Sound [31]. By current taxonomic definitions, individuals from both populations are members of a single species: their 18s rDNA gene sequences are identical, and there is no variation in the patterns of the silica cell wall, which are used traditionally to delineate species [30,31,33]. There are, however, differences between individuals from the two populations. Field isolates from population 1 are smaller than those from population 2 and the peaks of their blooms are temporally separated; this separation is differentially correlated to *in situ *silicic acid concentration and daily light exposure [30,31]. Even though both populations can be found in the water column at the same time, reproduction between them is limited, as evidenced by high F_ST _values (0.286) [30], which are consistent with the presence of cryptic species [35].

The observed differences in cell size and the limited gene flow between populations 1 and 2 of *Ditylum brightwellii *in the field led us to test the hypothesis that a difference in DNA content is associated with differences in acclimated growth rates and cell size separating the populations. Laboratory tests were performed within the context of two geographic scales, locally within Puget Sound, and globally, including *D. brightwellii *from New Zealand.

## Results

**Figure 1:**
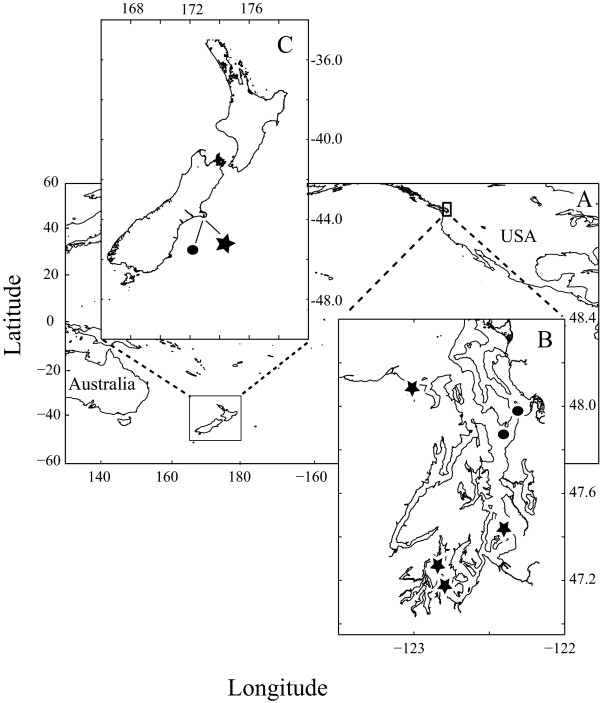
**Sampling locations from which clones were isolated**. A) Plan view of the Pacific Ocean with insets for: B) Puget Sound and C) New Zealand's Akaroa Harbour. Stars and circles represent sampling sites for clones isolated in 2006 and 2007, respectively.

Thirty-two isolates from Puget Sound, WA, USA and Akaroa Harbour, New Zealand (Fig. 1) were assigned to one of two *Ditylum brightwellii *populations based on the seven informative sites that distinguish the two ITS1 type-sequences [30]. The ITS1 sequences from all 10 New Zealand clones and 11 Puget Sound clones were identical to the type-sequence for population 1 (ITS1-1) from Puget Sound, including the seven informative sites, and were assigned to population 1. The ITS1 sequences for nine Puget Sound clones were identical to the ITS1-2 type sequence, including all of the informative sites, and were assigned to population 2. The ITS1 sequences for two Puget Sound clones were identical to the ITS1-2 type sequence at six of the seven informative sites. The ITS1 sequence of these two clones was polymorphic (C/T) at the fourth informative site, which was previously determined to have low-frequency variation between C and T and is therefore informative only if sequence at the other positions is known [31]. These two clones were also assigned to population 2.

**Figure 2:**
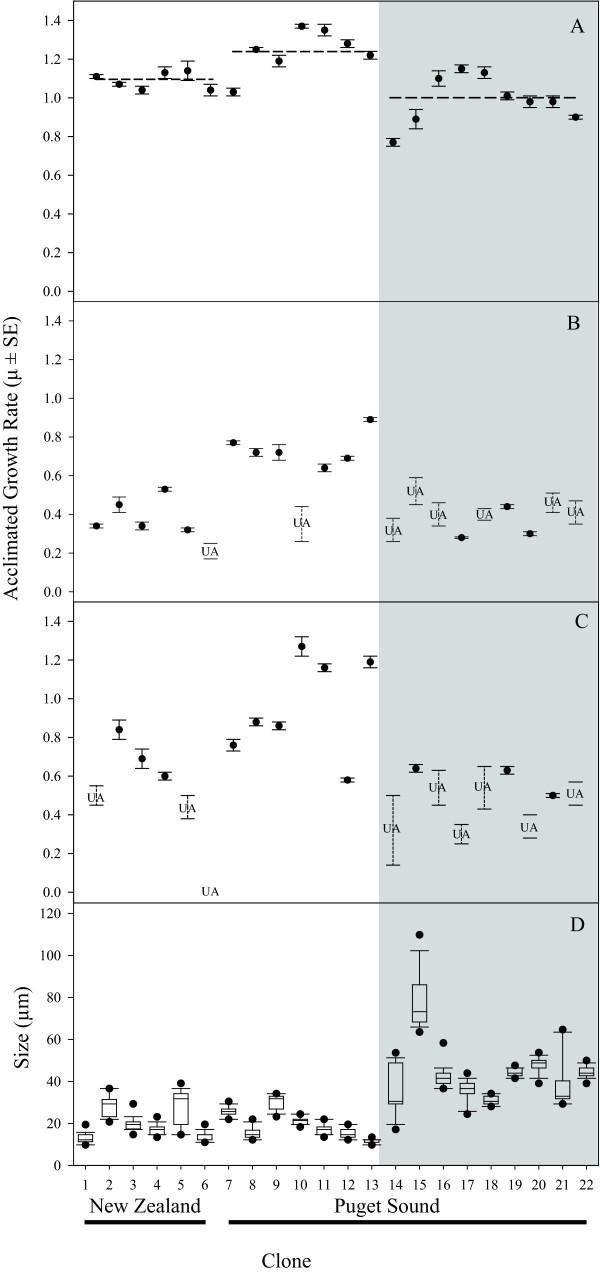
**Acclimated growth rates (A -C) and size distributions (D) of *Ditylum brightwellii***. Growth conditions: A) 110 μmol photons m^-2 ^s^-1^; 16:8 L:D; B) 115 μmol photons m^-2 ^s^-1^; 24 hour light; C) 60 μmol photons m^-2 ^s^-1^; 24 hour light. For acclimated clones, the mean growth rate ± standard error is provided. UA denotes clones unable to acclimate and the associated error bars indicate the range in growth rates. D) Boxplot parameters for the distributions of cell width (μm): bar = median; box = 1^st ^and 3^rd ^quartiles; whiskers = 10^th ^and 90^th ^percentiles; filled circles = outliers. N = 120 cells per clone. In all panels, white regions represent population 1 clones and grey regions represent population 2 clones.

Growth rates varied among clones within each of the three groups (New Zealand and Puget Sound populations 1 and 2), but among-group differences in growth rates were greater (Fig. 2A-C). When clones were maintained on a light:dark cycle, the mean growth rate of clones from Puget Sound population 1 (1.24 ± 0.11 d^-1^) was significantly faster than the mean growth rates of clones from either New Zealand (1.09 ± 0.04 d^-1^) or Puget Sound population 2 (0.99 ± 0.13 d^-1^) (ANOVA: F = 11.18, *p *= 0.001; Fig. 2A). Population 2 clones had the greatest range of growth rates, which included the slowest growing clones of either population, but the average growth rates of population 2 and New Zealand clones were not significantly different (Fig. 2A). Under conditions of continuous light, a majority of the population 2 clones failed to acclimate to the conditions within the eight week duration of each experiment (Fig. 2B, C). These un-acclimated clones grew, but no set of three consecutive transfer cultures grew at the same rate. In contrast, a majority of the population 1 clones were able to acclimate to growth under continuous light.

Cell widths were measured for clones included in the light:dark treatment of the growth rate experiments. Population 1 clones from New Zealand and Puget Sound had the same modal cell width of 14.6 μm (Fig. 2D). Population 2 clones from Puget Sound had a modal cell width of 43.9 μm, and were significantly larger than population 1 clones (Mann-Whitney U test; U = 29,672; *p *= 0.000), although individual clones from both populations did have overlapping size ranges (Fig. 2D).

**Figure 3:**
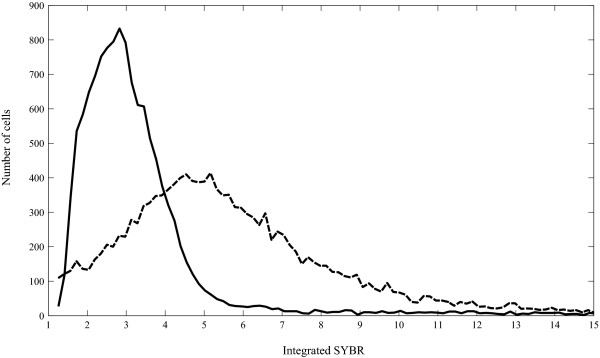
**Linearly calibrated integrated SYBR signals of two clones of *Ditylum brightwellii***. Population 1 (solid line) and population 2 (dashed line) are represented by histograms each consisting of 200 bins of equal size.

**Figure 4:**
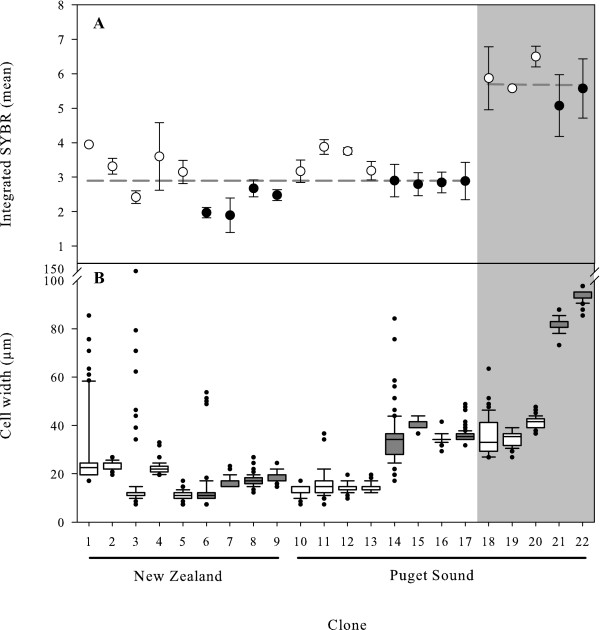
**Relative genome sizes (A) and cell size distributions (B) of *Ditylum brightwellii *from two populations**. A) The mean mode of the linearly calibrated integrated SYBR signal is given for clones collected in 2006 (white circles) where the whiskers represent the actual values of each duplicate, and clones collected in 2007 (black circles) where the whiskers represent the standard deviation of the triplicate samples. B) Size distributions of the cell width (μm) in each clone at the time of the flow cytometry measurements. N = 100 cells per clone. Boxplot parameters: bar = median; box = 1^st ^and 3^rd ^quartiles; whiskers = 10^th ^and 90^th ^percentiles; filled circles = outliers. White boxes represent 2006 clones and grey boxes represent 2007 clones. White panel-regions represent population 1 clones and grey panel-regions represent population 2 clones.

The relative difference in DNA content between the populations of *Ditylum brightwellii *was determined using flow cytometry to measure the integrated SYBR GREEN I fluorescent signal of 12 clones from the growth rate experiments and 10 fresh isolates. The resulting distributions of DNA content were unimodal regardless of which clone was analyzed, suggesting that when grown exponentially on a 16:8 light:dark cycle all clones had progressed similarly through the cell cycle and were sampled while the majority of cells were in G1. The DNA distributions of single clones from population 1 and 2 were significantly different from each other (Fig 3; Mann-Whitney U test, U = 3 × 10^7^, *p *= 0.000). To diminish the potential occurrence of multi-modality around the peaks of the distributions, non-uniform quantization of the signal, rather than traditional histograms, was used for subsequent analyses. The non-quantized value is termed the mode. Both methods of analysis resulted in distributions centered on single values (the modes) interpreted as the diploid DNA content of G1 cells. There was no indication of a second mode representing G2+M cells (e.g. Fig. 3). The DNA content per cell was not significantly different among individual clones within a population; the mean integrated SYBR signals of population 1 clones were 2.72 ± 0.78 and 3.11 ± 0.50, in relative units, for New Zealand and Puget Sound isolates, respectively. In contrast, the per cell DNA content of clones from population 2, 5.65 ± 0.82 relative units, was significantly greater than that of clones from population 1 (Fig. 4A; ANOVA: F = 73.29, *p *= 0.000). The ratio of the relative DNA content between populations 1 and 2 was 1.94 ± 0.74.

Clones associated with the DNA content experiments were isolated 18 months apart and cultured without controlling for size increases or decreases inherent in the diatom life history. Distributions of cell width were pooled for clones within each population, and the resulting two distributions were significantly different (Mann-Whitney U test, U = 63,932, *p = *0.000). Population 2 cells, which have a two-fold larger DNA content, tended to have greater minimum and maximum widths than cells from population 1 (Fig. 4B). A clear correlation between genome size and cell size is obscured by the overlap of cell width distributions of individual clones from each population (Fig. 4B). For example, clones 14-17 from Puget Sound population 1 and clones 18-20 from population 2 are of similar size, yet they have distinct genome sizes representative of their population of origin (Fig. 4A and 4B).

## Discussion

Diatoms are a relatively young, but diverse, group of eukaryotic micro-organisms that arose approximately 250 million years ago [36]. The genomes of diatoms appear to be highly flexible and evolve rapidly with respect to their size and gene content, which allows for ecological differentiation [14,37-40]. Here we present evidence that genomic flexibility underlies the recent divergence of two closely related populations of *Ditylum brightwellii*, distinguished from each other by a two-fold difference in DNA content. This difference in genome size appears to be stable regardless of the amount of time isolates from the two populations have been maintained as laboratory cultures.

The DNA content of *D. brightwellii *was previously estimated to be 12.9 pg per cell [21], which is the equivalent of 12.6 gigabases of DNA (assuming 980 MB pg^-1^;[41]) distributed amongst anywhere from 12-50 chromosomes in each diploid cell [42,43]. At least part of the explanation for the wide range in chromosome number is because karyotyping of diatoms is complicated by the presence of a rigid silica cell wall that prevents the consistent flattening of cells required to spread the condensed chromosomes apart for accurate enumeration [44]. We instead modified a flow cytometer to analyze thousands of the *D. brightwellii *cells that are up to 150 μm in width to gain an accurate estimate of the range of relative genome sizes for cultured isolates from Puget Sound, WA and from New Zealand. All distributions of DNA content were unimodal with a skew (right hand tail) towards higher DNA content. Actively dividing cells appear to spend the greatest proportion of their cell cycle in phases G1, forming the peak (or mode) of the distribution, and S, represented by cells under the tail. *Thalassiosira weissflogii*, a diatom with a six to twelve-fold smaller genome relative to *D. brightwellii *(Koester et al. unpublished data), spends a third of its cell cycle in S phase when maintained in continuous light and otherwise optimal growth conditions [45], suggesting that the S phase of the much larger genome of *D. brightwellii *represents an even greater proportion of the cell cycle. The level of experimental replication and consistency of the time of day that the cultures were collected and preserved argues strongly that cell cycle dynamics were comparable for clones of both populations, with few cells residing in the G2+M phase.

The most parsimonious explanation for the observed two-fold difference in DNA content between the two populations of *D. brightwellii *is a whole genome duplication event occurring within cells of population 1 creating a polyploid lineage that ultimately gave rise to population 2 cells. Mitotic and meiotic failures are potentially important and immediate mechanisms contributing to DNA content differences among diatom lineages, assuming that cells survive the initial mutation and are able to propagate asexually. Triploid and tetraploid zygotes have been observed in five genera of pennate diatoms [46-50], and non-disjunction of spermatocytes during meiosis was observed in the same species as stable polyploids [14]. Alternatively, genome size may increase via aneuploidy, gene duplication, or the rapid proliferation of transposable elements. Transposable elements were previously proposed as a primary mechanism of genome size expansion in diatoms [39] because there was no evidence of whole genome or segmental duplications in either of the two diatoms with completed genome sequences, yet both contained long-terminal-repeat retrotransposons [39,51].

The relationship between genome size and cell size spans five orders of magnitude for both factors and is a nearly universal trend in eukaryotes [20]. Diatoms, however, are unique in that cell-wall structure causes size reduction during asexual reproduction and different cells within a clone may have vastly different sizes; therefore, a clear correlation between cell size and genome size among closely related species with genomes of similar size is unlikely to be found. Nevertheless, there are indications that genome size influences the minimum size of *Ditylum brightwellii *clones. The appearance of large cells in clones dominated by small sizes suggests that the majority of population 1 was within the sexually inducible size range for *D. brightwellii *[52], and that minimum cell sizes were likely being approached by all of the clones isolated in 2006 and maintained in culture for 18 months. It is notable that the smallest cell sizes found in clones of population 2, which has the larger DNA content, tended to be larger than the smallest cell sizes found in clones from population 1. Most importantly, with regard to genome size and cell size, the diploid genome size is consistent among clones within a population, regardless of the variation in cell size caused by life history constraints.

Similar to traditional common garden experiments, our growth rate experiments tested for genetic differences among clones of *Ditylum brightwellii *from two populations, differentiated by genome size, and two groups of clones from population 1 represented by different geographic origins. The differences in growth rates between groups, populations 1 and 2 and New Zealand versus Puget Sound population 1 isolates, were greater than any within group variability. Growth rate variability may occur at the extremes of cell size within a clone [53,54], but those effects would be masked in our study by cell size variability within each clone and diminished by the large difference in growth rates between the groups. Population 2 clones grew more slowly than population 1 clones from the same region, consistent with the expectation that cells with larger cell sizes and larger genomes will have slower growth rates. However, population 1 clones from New Zealand grew more slowly than population 1 clones from Puget Sound, suggesting that other genetic factors are also responsible for setting rates of growth. New Zealand clones are likely adapted to oceanic conditions that are distinct from the estuarine waters of Puget Sound.

Rapid selection for cell and/or genome size among diatoms is indicated in the fossil record and appears to be associated with climate variability [55]. Large cells (> 100 μm diameter) of the morpho-species *Azpeitia nodulifera *(A. Schmidt) Fryxell and Sims (previously described as *Coscinodiscus nodulifer *Schmidt) intermittently enter and exit the sedimentary record, normally dominated by cells 40 μm in diameter, over the course of thousands of years [56,57]. The lack of variability in morphology and the 18S rDNA gene between the two populations of *Ditylum brightwellii *suggests that the genome duplication event was recent and rapid. Genomic plasticity has likely contributed to speciation among diatoms and may be an important factor in the adaptation of diatoms to future ocean conditions.

## Conclusions

The majority of phytoplankton have haplontic life histories [58]; therefore, a transition to a stable duplicated genome and a diplontic life history are likely at the root of the diatoms, the only phytoplankton group whose membership is diplontic. The propensity for further duplications may be a key mechanism of speciation among diatoms. Speciation is best identified by using a suite of divergent traits including reproductive isolation, morphology, ecological and physiological differences facilitated by genetic divergence. A high F_ST _value between the two populations of *Ditylum brightwellii *already indicated that that the process of reproductive isolation was underway, and that these two populations could represent separate species [30]. Duplicated genes, arising from the hypothesized whole genome duplication between the populations, may be released from selective pressures allowing for mutations that may be masked until environmental regimes change and they provide an adaptive advantage to the cell [59]. The apparent localization of population 2 to Pacific Northwest waters appears to reflect a recent divergence of the populations initiated by a whole genome duplication event. Population 1 and 2 most likely represent cryptic species in which interbreeding is greatly reduced and phenotypic differentiation is enhanced. In conjunction with previous work on genome size variation in diatoms, these results suggest that polyploidization is an active mechanism contributing to the diversification and speciation of marine diatoms.

## Methods

### Cell isolation and culturing

Single cells of *Ditylum brightwellii *were isolated from Puget Sound, Washington, USA and from the mouth of Akaroa Harbour, New Zealand during the spring and summers of 2006 and 2007 (Fig. 1). Thirty-two clonal, non-axenic cell lines were obtained by micropipetting individual cells through three aliquots of sterilized seawater into 0.5 ml f/10 medium [60] in a 48-well plate (Costar, Corning, NY). After one week, each clone was transferred to 25 ml f/2 medium and maintained as stock cultures at 13°C, with an irradiance of approximately 40 μmol photons m^-2 ^s^-1 ^and a photoperiod of 16:8 h light:dark.

### DNA sequencing

Fifty to 100 ml of clonal culture were filtered onto 25 mm, 5 μm pore size, polycarbonate membrane filters (Millipore) for DNA extraction using either the DNeasy Plant Mini Kit (Qiagen) or the Easy-DNA Kit (Invitrogen), following manufacturer instructions. The internal transcribed spacer sequence 1 (ITS1) was polymerase chain reaction (PCR)-amplified with primers 1645F and Dit5.8sR as described in [30]. Products from six amplification reactions were pooled and purified in one of two ways. The pooled PCR product was either directly purified using the High Pure PCR Product Purification Kit (Roche Applied Science) or electrophoresed in 1% agarose gels and bands of the appropriate size were excised and extracted from the agarose with the QIAquick Gel Extraction Kit (Qiagen). The resulting fragments were sequenced using primers 1645F and Dit5.8sR with the DYEnamic ET Terminator Cycle Sequencing Kit (GE Healthcare Bio-sciences Corp., New Jersey) and analyzed on a MegaBACE 1000 automated sequencer (GE Healthcare Biosciences Corp., New Jersey). Sequences were assigned to a population by aligning them to two type-sequences [Genbank: DQ329268] (population 1; ITS1-1) and [Genbank: DQ329270] (population 2; ITS1-2). Genbank accession numbers for ITS1 sequences from our study are [Genbank: GQ370472-GQ370503].

### Growth rate and size

Acclimated growth rates were determined using semi-continuous batch cultures [61] of the clones isolated in 2006 plus one clone from population 1 isolated in 1997 and one from population 2 isolated in 1998 from Puget Sound by [32,33]. The ITS1 sequences of the latter two clones were confirmed in this study. In total, six isolates from New Zealand, seven from Puget Sound population 1, and nine from Puget Sound population 2 were grown at 13°C under three different light conditions: continuous light of 60 and 115 μmol photons m^-2 ^s^-1^, and a 16:8 h light:dark cycle of 110 μmol photons m^-2 ^s^-1^. The 60 μmol photons m^-2 ^s^-1 ^continuous light experiment was completed prior to the other two, which were run simultaneously in separate incubators. Growth rates were determined by measuring chlorophyll *a *fluorescence daily with a Turner 10-AU Fluorometer (Sunnyvale, CA) and verified for a subset of clones by performing daily cell counts (data not shown). Acclimated growth rates of each clone were defined as the specific growth rates of cultures that were not significantly different over three consecutive transfers (ANCOVA; [62]); the common slope and associated standard error are reported as the acclimated growth rate.

Cell size was measured at two discrete times, once at the conclusion of the growth rate experiments (from the light:dark treatment) and once in conjunction with the DNA content experiments. Cells were preserved in a 1% final concentration each of formaldehyde and glutaraldehyde buffered with sterile f/2 medium. Cell width is the dimension of size reduction in *Ditylum brightwellii*; therefore, widths were measured in girdle view, perpendicular to the pervalvar (long) axis at the widest point, at 400× magnification using a Nikon Eclipse TS100 inverted microscope equipped with an ocular micrometer. Differences in size and growth rates among populations were analyzed using the statistics package SPSS (SPSS, Inc., Chicago, IL).

### Relative genome size

Relative DNA content (diploid genome size) was measured using flow cytometry for 12 clones isolated in 2006 and maintained in culture for 18 months and 10 clones isolated in 2007 approximately six months and six weeks, respectively, prior to measurement. One hundred ml of each clone were grown in a 16:8 h light:dark cycle with 110 μmol photons m^-2 ^s^-1 ^at 13°C. Clones were harvested in mid-exponential phase, half way through the light cycle, and concentrated by centrifugation (15 min at 1700 - 2000 × g). The pellet was suspended in 15 ml of 100% methanol at 4°C for 48 h to extract chlorophyll *a *[45]. Samples were centrifuged and washed twice with 4 ml phosphate buffered saline (PBS; 137 mM NaCl, 2.7 mM KCl, 10.4 mM Na_2_HPO_4_·H_2_O, 1.8 mM KH_2_PO_4_, pH = 7.4) before being resuspended in 3 ml PBS and treated with 30 μl RNase A (ca. 30 mg ml^-1^; R4642; Sigma-Aldrich, St. Louis, MO) at 37°C for 60 min. The DNA was stained with SYBR GREEN I (Invitrogen), at 1× final concentration, for at least 20 min. Fluorescent, 1 μm latex beads (Polysciences, Warrington, PA) were added as standards to each sample. Stained samples were kept on ice in the dark until run on the flow cytometer. Each clone of a sampling set (2006 or 2007) was analyzed on a given day, and replicate clonal samples were analyzed on separate days. Clones were grown and processed independently for replicate measurements; duplicate and triplicate measurements of relative genome size were made for 2006 and 2007 isolates, respectively. Mean sample sizes ranged from 8400 - 24,000 cells per clone per replicate.

An Influx Cell Sorter (BD Biosciences, San Jose, CA) was modified to include a 500 μm sample line, and a 200 μm nozzle producing a sample stream intercepting a 300 mW, 457 nm laser focused to 20 μm. A 10× objective lens and position sensitive detectors [63] allowed for the detection of the SYBR signal (530/20 nm bandpass filter), which was processed through an electronic integrator that produced a 16-bit data value (BD Biosciences, San Jose, CA).

Integrated SYBR signals for each cell of a single clone were usually unique; therefore, a central tendency, henceforth referred to as the mode, of the integrated SYBR signal was determined for each clone using a custom MATLAB script (MathWorks, Natick, MA). Non-uniform quantization was performed on the integrated SYBR signal for each replicate sample such that bins were variable in width. The number of cells in each bin was constant (160 cells bin^-1^) and optimized by choosing a cell number that minimized the mean square error of the integrated SYBR values within each bin and the number of equally narrow bins. The mean value of the integrated SYBR signal from the narrowest bin, which represented the greatest concentration of cells within the smallest range of integrated SYBR signals, was taken as the mode. In the minority of cases in which two nearby bins were equally narrow, the mean of the range of the two bins was calculated as the mode. Bimodality would be indicated by equally narrow bins occurring at a distance apart.

The integrator was calibrated for linearity by collecting the integrated fluorescent signals of beads (1 μm) and their doublets as the gain was increased by 2× intervals across the dynamic range of the integrator. The relationship between the linear input values of the beads and their integrated SYBR signal was determined to be of the form f(x) = A^bx^, where f(x) = linear value, A = 0.6445, b = 5.99 × 10^-5^, and x = integrated SYBR signal yielded the best fit (SSE = 1.397; R^2 ^= 0.9972; CurveFit Toolbox, MATLAB). Modal values of the integrated SYBR signal were applied to this equation with the linear output reported here.

## Authors' contributions

JAK participated in and implemented all levels of this study including experimental design, statistical analysis and drafting of the manuscript. JS executed the design, development and operation of the flow cytometer. PvD participated in preliminary experiments, final experimental design and drafting of the manuscript. EVA participated in experimental design and drafting of the manuscript. All authors have read and approve of the final manuscript.
